# Methylglyoxal products in pre-symptomatic type 1 diabetes

**DOI:** 10.3389/fendo.2023.1108910

**Published:** 2023-01-19

**Authors:** Sarah C. Shuck, Peter Achenbach, Bart O. Roep, John S. Termini, Carlos Hernandez-Castillo, Christiane Winkler, Andreas Weiss, Anette-Gabriele Ziegler

**Affiliations:** ^1^ Department of Diabetes and Cancer Metabolism, City of Hope, Duarte, CA, United States; ^2^ Institute of Diabetes Research, Helmholtz Munich, German Center for Environmental Health, Munich, Germany; ^3^ Forschergruppe Diabetes, School of Medicine, Klinikum rechts der Isar, Technical University Munich, Munich, Germany; ^4^ Forschergruppe Diabetes e.V. at Helmholtz Munich, German Research Center for Environmental Health, Munich, Germany; ^5^ Department of Internal Medicine, Leiden University Medical Center, Leiden, Netherlands; ^6^ Department of Molecular Medicine, City of Hope, Duarte, CA, United States

**Keywords:** advanced glycation end products, methylglyoxal, biomarker, RNA adduct, type 1 diabetes

## Abstract

**Introduction:**

Progression to type 1 diabetes has emerged as a complex process with metabolic alterations proposed to be a significant driver of disease. Monitoring products of altered metabolism is a promising tool for determining the risk of type 1 diabetes progression and to supplement existing predictive biomarkers. Methylglyoxal (MG) is a reactive product produced from protein, lipid, and sugar metabolism, providing a more comprehensive measure of metabolic changes compared to hyperglycemia alone. MG forms covalent adducts on nucleic and amino acids, termed MG-advanced glycation end products (AGEs) that associate with type 1 diabetes.

**Methods:**

We tested their ability to predict risk of disease and discriminate which individuals with autoimmunity will progress to type 1 diabetes. We measured serum MG-AGEs from 141 individuals without type 1 diabetes and 271 individuals with type 1 diabetes enrolled in the Fr1da cohort. Individuals with type 1 diabetes were at stages 1, 2, and 3.

**Results:**

We examined the association of MG-AGEs with type 1 diabetes. MG-AGEs did not correlate with HbA1c or differ between stages 1, 2, and 3 type 1 diabetes. Yet, RNA MG-AGEs were significantly associated with the rate of progression to stage 3 type 1 diabetes, with lower serum levels increasing risk of progression.

**Discussion:**

MG-AGEs were able to discriminate which individuals with autoantibodies would progress at a faster rate to stage 3 type 1 diabetes providing a potential new clinical biomarker for determining rate of disease progression and pointing to contributing metabolic pathways.

## Introduction

Type 1 diabetes is a disease characterized by a myriad of causes including autoimmunity, beta-cell dysfunction, and metabolic dysfunction. Methylglyoxal (MG) is an abundant reactive electrophile produced from protein, fatty acid, and sugar metabolism, with levels ~4 µM/day in basal conditions (1). MG forms covalent adducts on DNA, RNA, and protein termed MG-advanced glycation end products (MG-AGEs). MG-AGEs are associated with type 1 diabetes and the risk of developing complications, but their ability to predict progression to clinical type 1 diabetes is not clear (2, 3). As MG is produced from a variety of metabolic pathways, MG-AGEs are proposed to provide a more comprehensive measure of metabolic changes associated with type 1 diabetes compared to hyperglycemia alone. Several previous studies show that children have elevated blood glucose levels long before the clinical manifestation of type 1 diabetes (4–6). We hypothesized that MG-AGEs may be associated with the clinical onset of type 1 diabetes and correlated with the rate of disease progression. We therefore investigated serum samples of children participating in the public health islet autoantibody screening study Fr1da (7).

## Methods

*N^2^*-carboxyethyl-guanosine (CEG) was quantified using liquid chromatography tandem mass spectrometry (LC-MS/MS) using isotopically labeled standards in the laboratories of S. Shuck and J. Termini through modification of a previously described method for *N^2^*-carboxyethyl-deoxyguanosine CEdG measurement (8). A total of 20 µl of serum was used. Samples were blinded so that the investigators did not know the group assignment as defined in **Table 1**. The limit of detection of this assay was 0.01 ng/mL and the limit of quantitation was 0.05 ng/mL. Technical variation between independent sample workups was <2%. A representative chromatogram of CEG and CEdG can be found in ESM **Figure 1**).

**Table 1:** Characteristics of study population.

	IAb neg	Stage1 -T1D	Stage 2 -T1D	Stage 3 -T1D
n	141	240	22	9
Age (years), (median, IQR)	4.6 (3.3-5.7)	4.3 (3.3-5.6)	4.3 (3.2-5.2)	4.8 (4.2-8.2)
Sex m/f	68/73	131/109	16/6	5/4
Number of pos IAb				
2 3 4	/ / /	82 76 82	4 12 6	1 3 5
CEG (pmol/ml) (median, IQR)	0.039 (0.020, 0.169)	0.043 (0.020, 0.185)	0.045 (0.023, 0.167)	0.019 (0.000, 0.030)
Developed Stage 3 T1D during FU	0	55	14	/

*The study included 271 children with 2 or more islet autoantibodies (IAA, GADA, IA-2A, or ZnT8A) and 141 islet autoantibody-negative children.

IAb, islet autoantibody; T1D, type 1 diabetes; IQR, interquartile range; m, male; f, female; FU, follow-up.

**Figure 1:**
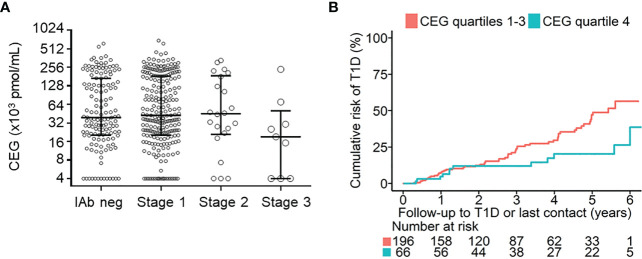
Relationship between CEG levels and islet autoantibody status, stage of pre-symptomatic type 1 diabetes (T1D), and risk of progression to clinical T1D (stage 3). **(A)** shows CEG levels for 141 islet autoantibody-negative children (IAb neg), 240 children with stage 1 T1D, 22 children with stage 2 T1D, and 9 children with stage 3 T1D. Median (IQR) values are shown for each group. **(B)** shows the cumulative risks of developing stage 3 T1D in children with stage 1 or 2 T1D for children with CEG levels in the highest quartile (Q4) of measurements (blue line) compared with children with lower CEG levels (Q1-Q3; red line) (P=0.001; log-rank test). Follow-up begins at initial staging by OGTTs. The number of children at risk is shown below each time point..

Samples from children who participated in the Fr1da public health screening for islet autoantibodies were studied, including 271 with 2 or more islet autoantibodies (i.e. autoantibodies to insulin [IAA], glutamic acid decarboxylase [GADA], insulinoma antigen-2 [IA-2A], or zinc transporter 8 [ZnT8A]), and 141 who tested islet autoantibody negative (7, 9). All children with islet autoantibodies had participated in a staging visit in which an Oral Glucose Tolerance Test (OGTT) was performed resulting in 240 children diagnosed with stage 1, 22 with stage 2, and 9 with stage 3 type 1 diabetes (**Table 1**). Stages of type 1 diabetes were classified according to the consensus criteria of the JDRF, Endocrine Society and ADA of 2015, as previously described (4). Stage 1 type 1 diabetes was defined as positivity for multiple islet autoantibodies and normal glucose tolerance based on OGTT results. Stage 2 type 1 diabetes was defined as positivity for multiple islet autoantibodies accompanied by dysglycaemia based on OGTT results (fasting plasma glucose of 6.1– 6.9 mmol/l [110–125 mg/dl] or impaired 2 -h plasma glucose of 7.8–11.0 mmol/l [140–199 mg/dl], and/or plasma glucose ≥ 11.1 mmol/l [200 mg/dl] at 30, 60 or 90 minutes). Stage 3 type 1 diabetes was defined based on ADA criteria (10). The staging sample was used for measurement of CEG concentrations. Written informed consent was obtained from the children’s parents or legal guardians. Children were prospectively followed for the development of stage 3 type 1 diabetes as previously described (7). CEG concentrations were compared between groups by Mann Whitney test. Correlations of CEG with HbA1c or age were calculated by Spearman test. The progression to clinical diabetes was assessed using the Kaplan–Meier time-to-event method. Children were censored when they developed stage 3 type 1 diabetes or reached the date of their final contact to ascertain diabetes status. Between-group comparisons in the Kaplan–Meier analyses were performed using the log rank test. For this analysis, CEG values were categorized into quartiles (Q). The survival analyses and plots were prepared using R version 4.0.2 and the package ‘survival’ v3.2-7.

## Results

We first asked whether CEG levels were different between islet autoantibody-negative and islet autoantibody-positive children and whether CEG levels were related to the pre-symptomatic stage of type 1 diabetes. Median CEG levels did not differ between the 141 islet autoantibody-negative children (0.039 pmol/ml [interquartile range; 0.020, 0.169]) and the 271 islet autoantibody-positive children (0.042 pmol/ml [0.020, 0.181]; *P* = 0.94) or between islet autoantibody-negative children and children with stage 1 or stage 2 type 1 diabetes (**Supplementary Figure 2A** and **Table 1**). Children with stage 3 type 1 diabetes had slightly lower median CEG levels (0.019 pmol/ml [0.000, 0.030]; *P* = 0.05 compared to islet autoantibody-negative group), but the number of cases in this group was small (n = 9). CEG concentrations did not significantly correlate with HbA1c (*r* = -0.1204, *P* = 0.05) or age (*r* = 0.0345, *P* = 0.48) (ESM **Supplementary Figures 2A, B**) at the time of measurement. During prospective follow-up, 69 of 262 islet autoantibody-positive children with stage 1 or 2 type 1 diabetes progressed to stage 3 type 1 diabetes (**Table 1**). In these children, disease progression from stage 1 or stage 2 to stage 3 type 1 diabetes was significantly lower in children with CEG levels in the highest quartile (Q4) of measurements (20.3% [95% CI; 7.6 – 31.2] by 5 years follow-up vs. 47.1% [35.7 – 56.5] in children with CEG levels in Q1-Q3; *P* = 0.001) (**Supplementary Figure 2B**) or compared to children with CEG levels in Q1 (58.4% [95% CI; 36.1 - 72.6]; *P* = 0.014) (ESM **Supplementary Figure 3**). Similar results were obtained when children with stage 2 were excluded from the Kaplan-Meier analysis (Q4 16.1% [95% CI; 4.0 – 26.8] by 5 years follow-up vs. 43.1% [31.2 – 53.0] in children with CEG levels in Q1-Q3; P = 0.001 (ESM **Supplementary Figure 4**), and 55.4% [31.2 – 71.0] in children with CEG levels in Q1 (P = 0.011).

## Discussion

Our results reveal the first association of MG-AGEs with progression to clinical type 1 diabetes (stage 3). Although we measured a panel of MG-AGEs, only the RNA adduct CEG was observed in serum (data not shown). The observation that MG-AGEs are negatively associated with progression to clinical type 1 diabetes may result from increased urinary excretion of these adducts resulting in lower serum levels or intracellular accumulation of adducts. Defining this relationship requires additional experimentation in human, mouse, and *in vitro* models, which we are currently investigating. Alternatively, counteractive mechanisms may affect the metabolic pathways generating MG-AGEs in an attempt to slow the latest stages in disease progression before clinical manifestation. Exploring these possibilities will help elucidate the extent to which CEG may be a driver of beta-cell destruction. One of the strengths of our study was the observation of a significant association of RNA MG-AGEs with the rate of type 1 diabetes progression as well as the large number of children with early disease stages investigated. RNA damage has not been significantly explored as a biomarker of disease but has emerging importance both as a marker and potential driver of disease. Furthermore, CEG may have utility in biomarker panels that measure metabolic changes that occur prior to clinical type 1 diabetes diagnosis. Limitations of this study include a lack of longitudinal samples and no matched serum and urine samples to investigate how excretion influences serum levels of MG-AGEs. The current results provide a framework for additional investigations of this adduct in metabolic disease models, including as predictors of diabetic complications.

## Data availability statement

The raw data supporting the conclusions of this article will be made available by the authors, without undue reservation.

## Author contributions

SS and BR conceived the objective of this study. A-GZ is the principal investigator of the Fr1da study. A-GZ and PA were responsible for the study design. PA, CW, AW, SS, BR, and CH-C contributed substantially to data acquisition and analysis. A-GZ, PA, SS, and BR drafted the manuscript. SS takes responsibility for the integrity of the work as a whole. All authors contributed to the article and approved the submitted version.
