# Supplementary material for: Methylglyoxal products in pre-symptomatic type 1 diabetes
Source: Front Endocrinol (Lausanne). 2023 Jan 19;14:1108910. doi: 10.3389/fendo.2023.1108910 (PMC9892703; doi:10.3389/fendo.2023.1108910)
Supplement: Supplementary file 1 [file DataSheet_1.pdf]

Supplementary Material

Methylglyoxal products in pre-symptomatic type 1 diabetes

Sarah C. Shuck[1*], Peter Achenbach[2,3,4], Bart O. Roep[5], John S. Termini[6], Carlos Hernandez-Castillo[1], Christiane Winkler[2,4], Andreas Weiss[2,4], Anette-Gabriele Ziegler[2,3,4]

[1]Department of Diabetes and Cancer Metabolism, City of Hope, Duarte, CA, USA

[2] Institute of Diabetes Research, Helmholtz Munich, German Center for Environmental Health, Munich, Germany

[3] Forschergruppe Diabetes, School of Medicine, Klinikum rechts der Isar, Technical University Munich, Munich, Germany

[4] Forschergruppe Diabetes e.V. at Helmholtz Munich, German Research Center for Environmental Health, Munich, Germany

[5] Department of Internal Medicine, Leiden University Medical Center, Leiden, Netherlands

[6] Department of Molecular Medicine, City of Hope, Duarte, CA USA

*** Correspondence:**
Sarah C. Shuck
sshuck@coh.org

1.1 Supplementary Figures

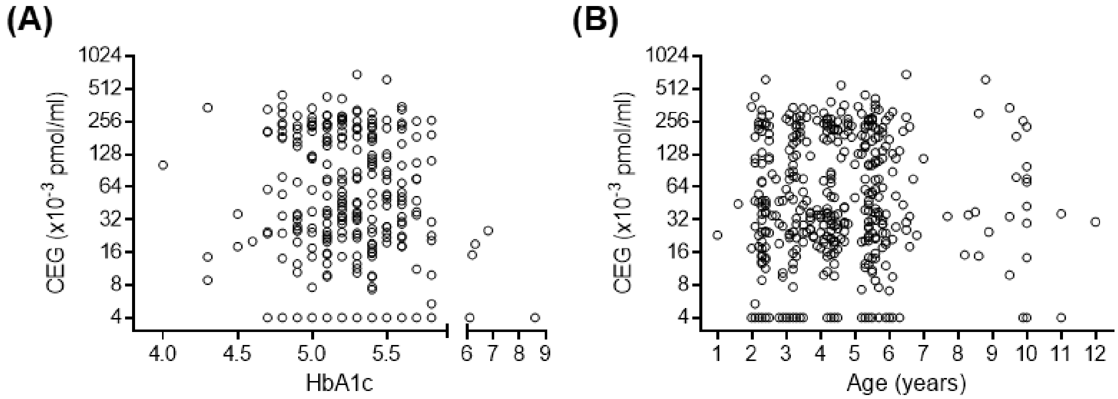

Supplementary Figure 1. Relationship between CEG levels and HbA1c and age. CEG levels are plotted against the HbA1c levels of 265 islet autoantibody-positive children (**A**) and the age of 412 islet autoantibody-positive or -negative children (**B**).

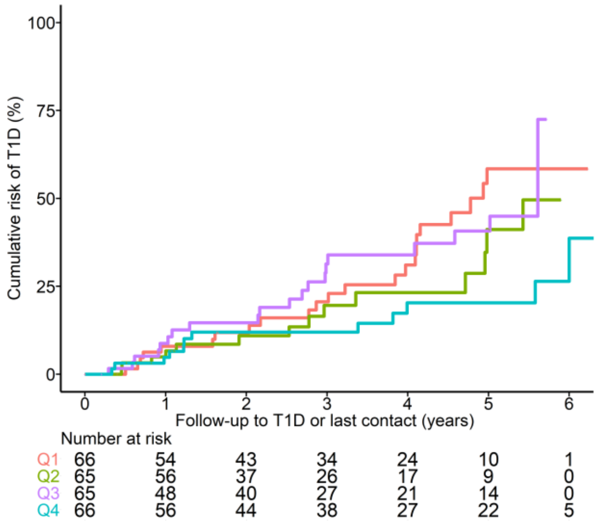

Supplemental Figure 2 The cumulative risks of developing stage 3 T1D in children with stage 1 or 2 T1D for children with CEG levels in the lowest/first quartile (Q1; red line) of measurements, the second quartile (Q2; green line), the third quartile (Q3; purple line), and in the highest/fourth quartile

(Q4; blue line). Follow-up begins at initial staging by OGTTs. The number of children at risk is shown below each time point.

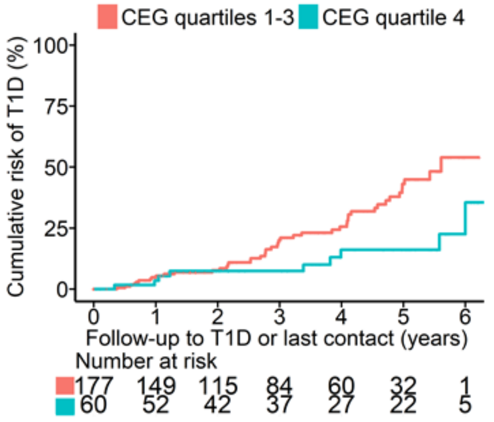

Supplementary Figure 3. The cumulative risks of developing stage 3 T1D in children with stage 1 T1D are shown for children with CEG levels in the highest quartile (Q4) of measurements (blue line) compared with children with lower CEG levels (Q1-Q3; red line) (P=0.001; log-rank test). Follow-up begins at initial staging by OGTTs. The number of children at risk is shown below each time point.

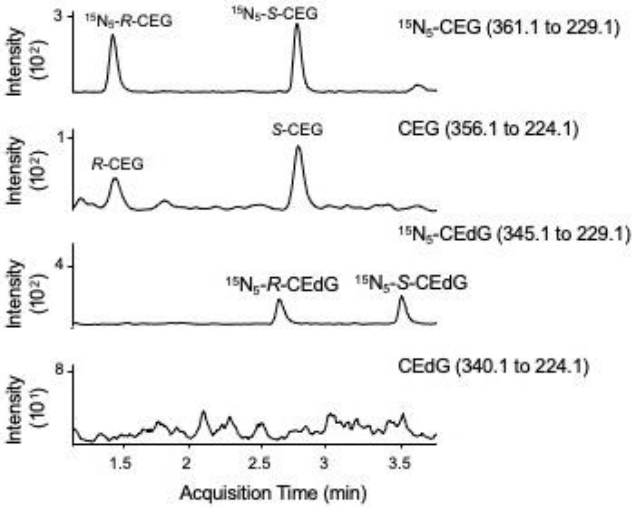

Supplementary Figure 4. Representative chromatogram of CEG and CEdG and $^{15}N_5$-isotopically labeled standards measured from human serum. CEdG and CEG were analyzed using LC-MS/MS with the mass transitions monitored in positive ion mode as indicated. Each analyte is present as *R*- and *S*-diastereomers, which are resolved chromatographically. The limit of detection was 0.01 ng/mL and the limit of quantitation was 0.5 ng/mL.
